# Developmental Toxicity of Chlorinated Polyfluorinated Ether Sulfonate (F-53B), a Perfluorooctane Sulfonate (PFOS) Alternative, in Embryos and Larvae of Blotched Snakehead (*Channa maculata*)

**DOI:** 10.3390/antiox15030368

**Published:** 2026-03-13

**Authors:** Yuntao Lu, Ziwen Yang, Yang Zou, Yueying Deng, Luping Liu, Jian Zhao, Qing Luo, Haiyang Liu, Shuzhan Fei, Kunci Chen, Yuandong Sun, Mi Ou

**Affiliations:** 1 School of Life and Health Sciences, Hunan University of Science and Technology, Xiangtan 411201, China; 17633537502@163.com (Y.L.); dyy12504@163.com (Y.D.); lluping0308@163.com (L.L.); 2 Key Laboratory of Tropical and Subtropical Fishery Resources Application and Cultivation, Ministry of Agriculture and Rural Affairs, Pearl River Fisheries Research Institute, Chinese Academy of Fishery Sciences, Guangzhou 510380, China; 17707276905@163.com (Z.Y.); zy20030430zy@163.com (Y.Z.); zhaojian@prfri.ac.cn (J.Z.); luoqing@prfri.ac.cn (Q.L.); hyliu@prfri.ac.cn (H.L.); feisz@prfri.ac.cn (S.F.); chenkunci@prfri.ac.cn (K.C.); 3 School of Fishery, Zhejiang Ocean University, Zhoushan 316022, China; 4 College of Fisheries and Life Sciences, Shanghai Ocean University, Shanghai 201306, China

**Keywords:** *Channa maculata*, F-53B, developmental toxicity, histopathology, oxidative stress, transcriptomics

## Abstract

Chlorinated polyfluoroalkyl ether sulfonate (F-53B), a common substitute for perfluorooctane sulfonate (PFOS), exhibits similar environmental persistence and bioaccumulation potential, raising concerns about its ecological and health impacts. However, comprehensive toxicological data remain limited for adequate environmental risk assessment. In this study, we evaluated the developmental toxicity of F-53B using embryos/larvae of a commercially important benthic fish, blotched snakehead (*Channa maculata*). Embryos (<1 h post-fertilization, hpf) were exposed to various concentrations of F-53B (0.002, 0.02, 0.2, and 2 mg/L) for 120 h. Exposure resulted in concentration-dependent adverse effects, including reduced hatching success, increased mortality, and morphological malformations (yolk sac edema, spinal curvature). Histopathological analysis revealed substantial hepatic injury (vacuolization, nuclear pyknosis) and intestinal damage (villi atrophy) at higher concentrations (0.2 and 2 mg/L). Mechanistically, F-53B induced oxidative stress through inhibition of superoxide dismutase (SOD) and catalase (CAT), depletion of glutathione (GSH), and elevated malondialdehyde (MDA). Additionally, the observed immune dysregulation was characterized by the up-regulation of pro-inflammatory cytokines, including interleukin 1β (*IL-1β*), interleukin 8 (*IL-8*), and tumor necrosis factor-α (*TNF-α*), consistent with activation of the TLR-MAPK signaling pathway, and coincided with a shift from metabolic adaptation to pronounced inflammation. These integrated findings indicate that F-53B impairs early development in *C. maculata* through pathways involving oxidative damage, tissue injury, and immune disruption. This underscores the ecological risk F-53B poses to aquatic organisms and highlights the need for more comprehensive environmental risk assessment.

## 1. Introduction

Perfluorooctane sulfonate (PFOS), a synthetic per- and polyfluoroalkyl substance (PFAS), has been widely used since the mid-20th century in industrial and consumer products such as textiles, electronics, and fire-fighting foams due to the exceptional stability conferred by its hydrophobic carbon-fluorine bonds and a hydrophilic sulfonate group [1,2]. This stability renders PFOS highly resistant to environmental degradation, such as photolysis, hydrolysis, and biodegradation, resulting in its persistence and widespread detection across multiple environmental compartments [3,4,5]. PFOS also bioaccumulates and biomagnifies through aquatic food webs, reaching higher-trophic organisms, including humans, and has been linked to various adverse health effects [6,7,8,9,10]. In response to these risks, China restricted the production, import, and use of PFOS in 2009 [11], which prompted the adoption of alternative compounds such as chlorinated polyfluoroalkyl ether sulfonate (Cl-PFESA, trade name F-53B) [12].

Structurally similar to PFOS, F-53B exhibits comparable chemical stability and hydrophobic/oleophobic properties [13] and has been employed as a substitute in chrome plating, textiles, and electronics [12,14]. Although commercially successful, F-53B received limited scientific attention until its first reported detection in environmental samples in 2013 [15]. Subsequent studies have detected F-53B in various environmental media, including chrome-plating wastewater (43~112 μg/L) [15], river (0.59~77 ng/L) [16,17], seawater (<7.85 ng/L) [18], river sediments (<7.2 ng/g) [16], and electroplating plant sludge (210 ng/g) [15]. Research in China’s Pearl River Basin reported concentrations of 0.74 μg/L in wastewater treatment plant influent and 0.22 μg/L in effluent, indicating that conventional treatment methods are largely ineffective at removing this compound [19]. Its detection in surface waters across multiple countries further suggests that F-53B is a potential global environmental contaminant [20].

F-53B exhibits high bioaccumulation potential and has been widely detected in aquatic organisms. In crucian carp (*Carassius carassius*), it accumulates in the blood, liver, and gonads [21]. In zebrafish (*Danio rerio*), it is present in muscle, ovaries, and embryos at 5 days post-fertilization [22]. Similarly, in the rare minnow (*Gobiocypris rarus*), F-53B has been detected in blood, liver, gonads, and gills [23]. It has also been detected in high-trophic-level Arctic mammals, such as the liver of polar bears (*Ursus maritimus*) and ringed seals (*Pusa hispida*) [24]. Human exposure is also documented, with F-53B identified in serum, urine, and breast milk at maximum concentrations of 5.04 μg/L, 2.86 μg/L, and 0.32 μg/L, respectively [25,26,27,28]. Notably, F-53B levels in breast milk can exceed those of PFOS [28]. Toxicological studies indicate that F-53B may be more biologically persistent and have a longer half-life than PFOS [29,30]. Substantial evidence indicates that such accumulation poses health risks. In *D. rerio* embryos, F-53B exposure reduces hatching rates, increases malformations, and induces oxidative stress along with thyroid, neurological, and immunological toxicity [14,31,32,33]. Similarly, in chicken (*Gallus gallus*), embryos treated with F-53B lead to decreased heart rates and significant liver enlargement [34]. In mice (*Mus musculus*), F-53B elevates reactive oxygen species (ROS) and apoptosis in pre-implantation embryos [35], disrupts neural development in differentiating embryonic stem cells [36], and impairs trophoblast function, resulting in delayed embryonic development [37]. Collectively, these findings indicate that F-53B disrupts early development across species, primarily through mechanisms involving oxidative stress and immune dysregulation.

Blotched snakehead (*Channa maculata*) is a benthic freshwater fish of high economic importance in Chinese aquaculture, prized for its rapid growth and high-quality flesh [38]. Its bottom-dwelling behavior increases the risk of exposure to sediment-accumulating pollutants such as F-53B. This study therefore investigates the effects of F-53B on the early embryonic and larval development of *C. maculata* by assessing mortality and malformation rates, and employing histological, biochemical, and transcriptomic analyses. The findings aim to elucidate the adverse impacts of F-53B on a commercially important fish, provide critical data for ecological risk assessment, and establish a scientific basis for environmental protection strategies concerning this emerging aquatic contaminant.

## 2. Materials and Methods

### 2.1. Ethical Approval

All animal procedures were approved by the Animal Ethics Committee of the Pearl River Fisheries Research Institute, Chinese Academy of Fishery Sciences (Approval No. LAEC-PRFRI-2024-08-04) and were conducted in accordance with relevant animal welfare guidelines.

### 2.2. Chemicals

F-53B (C_8_ClF_16_O_4_SK, CAS # 73606-19-6, purity ≥ 98%) was purchased from Shanghai Maikun Chemical Co., Ltd. (Shanghai, China). A stock solution (20 mg/mL) was prepared by dissolving 20 mg of F-53B powder in 100 μL of dimethyl sulfoxide (DMSO; MPBiomedicals LLC, Irvine, CA, USA) and diluting to 1 mL with deionized water. Assay kits for total protein (TP, A045-2), malondialdehyde (MDA, A003-1), glutathione (GSH, A006-2), superoxide dismutase (SOD, A001-3), and catalase (CAT, A007-1) were obtained from Nanjing Jiancheng Bioengineering Institute (Nanjing, China). All other chemicals and solvents, of analytical or high-performance liquid chromatography grade, were supplied by Sinopharm Chemical Reagent Co., Ltd. (Shanghai, China).

### 2.3. Experimental Fish and Design

Broodstock of *C. maculata* were maintained at the Fangcun Experiment Station of Pearl River Fisheries Research Institute in Guangzhou, China. Sexually mature individuals were selected for artificial reproduction following established protocols [39]. Healthy, newly fertilized embryos (<1 h post-fertilization, hpf) were used for exposure experiments. Embryonic developmental stages were determined according to the standard morphological criteria for *C. maculata*, and only embryos at the cleavage stage exhibiting normal morphology and clear cell division were selected.

For the exposure experiment, embryos were exposed to four nominal concentrations of F-53B: 0.002, 0.02, 0.2, and 2 mg/L. The lowest concentration (0.002 mg/L) was chosen to approximate environmentally relevant levels based on reported detections in surface waters near industrial areas (up to 0.001 mg/L) and in chrome plating wastewater (up to 0.112 mg/L) [40,41]. The intermediate concentrations (0.02 and 0.2 mg/L) were selected to bridge the gap between environmental levels and those previously reported to induce acute lethal effects in *D. rerio* embryos [31], thereby enabling the establishment of concentration–response relationships. The highest concentration (2 mg/L) was determined based on preliminary range-finding tests guided by studies on *D. rerio* embryos [14,42], in which complete mortality of *C. maculata* embryos occurred at 10 mg/L by 72 hpf and at 5 mg/L by 96 hpf. Since exposure to 2 mg/L induced significant phenotypic effects while allowing survival beyond 120 hpf, this concentration was selected as the highest treatment level. All treatment groups, including the control, contained a final DMSO concentration of 0.001% (*v*/*v*) to maintain solvent consistency. This concentration is well below the maximum recommended solvent concentration of 0.01% (100 μL/L) for medaka exposure studies, as specified in the OECD (2015) guidelines [43]. Furthermore, preliminary range-finding tests confirmed that 0.001% DMSO effectively solubilized F-53B without inducing observable toxicity in *C. maculata* embryos.

All treatments, including the control, were performed in triplicate (*n*= 3). For each replicate, 300 embryos were randomly placed in a 1-L glass aquarium containing the corresponding exposure solution, resulting in a total of 900 embryos per treatment group. Incubation was maintained at 26~28 °C under a 12 h:12 h light:dark cycle, with 50% of the test solution renewed every 24 h. Water quality parameters were maintained within standardized aquaculture requirements: pH 7.0~7.5, dissolved oxygen (DO) at 6~8 mg/L, ammonia nitrogen below 0.5 mg/L, nitrite below 0.01 mg/L, and water hardness 110~130 mg/L (as CaCO_3_). The exposure lasted 120 h. Dead embryos, identified by opacity, whitening, or cardiac arrest, were recorded and removed daily. Larvae malformations, such as pericardial oedema, yolk sac oedema, and spinal curvature, were examined and documented using a microscope (Nikon, Tokyo, Japan). To minimize observer bias, malformation scoring was independently conducted by two researchers blinded to treatment allocation, and treatment codes were revealed only after all data had been recorded. At 120 hpf, 48 larvae were sampled from each replicate per concentration. Of these, 5 larvae per replicate were fixed in 4% paraformaldehyde (PFA, BBI, Shanghai, China) for histological analysis. The remaining larvae were flash-frozen in liquid nitrogen and stored for subsequent transcriptomic, enzymatic, and gene expression analyses.

### 2.4. Histological Analysis

Whole larvae were fixed in 4% PFA for 24 h at room temperature, dehydrated through a graded ethanol series (70%, 80%, 90%, and 100%), cleared in xylene, and embedded in paraffin wax. Transverse sections (4 μm) were cut using a rotary microtome (Leica, Wetzlar, Germany), stained with hematoxylin and eosin (H&E), and examined under a light microscope (Nikon, Japan).

### 2.5. Enzyme Activity Assays

For each treatment, 15 larvae from each of three replicate aquariums were pooled to generate one composite sample, yielding three biological replicates (*n* = 3) per concentration. Pooled samples were homogenized in a 10-fold volume (*w*/*v*) of ice-cold physiological saline (0.9%), and the homogenate was centrifuged at 2500× *g* at 4 °C for 10 min. The supernatant was collected for biochemical assays. Levels of MDA (λ_max_ 532 nm) and GSH (λ_max_ 420 nm), as well as the activities of SOD (λ_max_ 550 nm) and CAT (λ_max_ 405 nm), were measured with commercial assay kits according to the manufacturer’s instructions. All enzyme activities were normalized to TP content, determined using a BCA protein assay kit (Thermo Fisher, Waltham, MA, USA). The units were expressed as follows: MDA in nmol/mg protein, GSH in μmol/g protein, SOD in U/mg protein, and CAT in U/mg protein. The detection limits were 0.5 nmol/mL for MDA, 0.3 nmol/L for GSH, 0.5 U/mL for SOD, and 0.2 U/mL for CAT. All sample measurements fell within the linear range of the standard curves.

### 2.6. Transcriptome Sequencing

For each concentration, 15 larvae from each of three replicate aquariums were pooled to form one composite sample, resulting in three biological replicates (*n* = 3) per treatment group. Total RNA was extracted from each composite sample using TRIzol reagent (Invitrogen, Waltham, MA, USA). RNA integrity, purity, and concentration were assessed using an Agilent 2100 Bioanalyzer (Agilent Technologies, Santa Clara, CA, USA) and a NanoDrop ND-2000 spectrophotometer (Thermo Scientific, Waltham, MA, USA). Subsequently, a sequencing library was constructed for each biological replicate using the Hieff NGS^®^ Ultima Dual-mode mRNA Library Prep Kit (Yeasen, Shanghai, China). In total, 15 libraries (three per treatment group) were sequenced on an Illumina NovaSeq 6000 platform (Guangzhou Kigio Biotechnology Co., Ltd., Guangzhou, China).

Raw reads were quality-checked with FastQC (v. 0.11.8). High-quality clean reads were aligned to the *C. maculata* reference genome (SRA Accession No. PRJNA730430) [44] using HISAT2 (v2.0.4). Gene expression levels were quantified as Transcripts Per Kilobase Million (TPM). Differential expression analysis was performed with DESeq2 package (v1.30.1), defining differentially expressed genes (DEGs) as those with |log_2_ (fold change)| > 1 and a false discovery rate (FDR) < 0.05. Gene Ontology (GO) and Kyoto Encyclopedia of Genes and Genomes (KEGG) enrichment analyses were performed using the OmicShare online cloud platform (www.omicshare.com/tools, accessed on 8 January 2026).

### 2.7. Quantitative Real-Time PCR (qRT-PCR)

To validate transcriptomic results, the expression of selected immune-related genes was analyzed by qRT-PCR. Composite samples (*n* = 3) were prepared by pooling 10 larvae from each replicate aquarium per concentration. Total RNA was extracted with TRIzol, and cDNA was synthesized using the PrimeScript™ RT Reagent Kit with gDNA Erase (Takara, Kusatsu-shi, Japan). Gene-specific primers (Table 1) were designed using Premier 5.0 or obtained from literature [45,46].

The target genes analyzed were interleukin 1β (*IL-1β*), interleukin 10 (*IL-10*), tumor necrosis factor-α (*TNF-α*), interleukin 8 (*IL-8*), interleukin 17 (*IL-17*), C-C chemokine receptor type 6 (*CCR6*), C-X-C chemokine receptor type 3 (*CXCR3*), and nuclear factor kappa B (*NF-κb*), with beta actin (*β-actin*) serving as the reference gene. Reactions were performed in triplicate on a StepOnePlus™ Real-Time PCR System (ABI, Foster City, CA, USA). Relative gene expression was calculated using the 2^−^^ΔΔCt^ method.

### 2.8. Data Analysis

Data are presented as mean ± standard deviation (SD). Differences between the control and treatment groups were evaluated using one-way analysis of variance (ANOVA) followed by Duncan’s multiple range test, which was selected for its sensitivity in detecting subtle differences between groups with relatively small sample sizes in this exploratory study. *p* < 0.05 was considered statistically significant. The median lethal concentration (LC_50_) was calculated using non-linear regression in SPSS (version 26.0). Concentration-response data were fitted to the “[log(agonist) vs. response (variable slope)]” model, which assumes a symmetrical sigmoidal curve. Model adequacy was assessed using *R^2^* value and visual inspection of the fitted curve. The LC_50_ was defined as the concentration causing 50% mortality, with 95% confidence intervals estimated by the software. All statistical analyses, including LC_50_ calculation, were performed using SPSS (version 26.0).

## 3. Results

### 3.1. Effects of F-53B on the Embryonic Development of C. maculata

**Figure 1:**
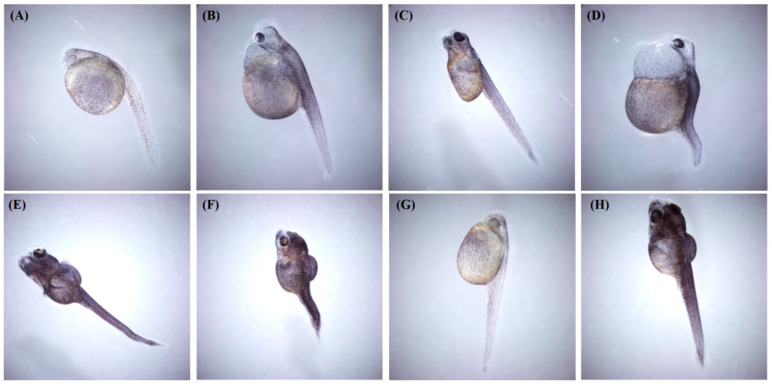
Teratogenic effects of F-53B on *C. maculata* larvae. (**A**) yolk sac edema, (**B**) pericardial and yolk sac edema, (**C**) abnormal yolk sac morphology, (**D**) co-occurring pericardial edema, yolk sac edema, and spinal curvature, (**E**) tail curvature, (**F**) tail and spinal curvature, and (**G**,**H**) normal larvae.

Embryonic and larval development of *C. maculata* was monitored for 120 h using an inverted microscope. Pericardial and yolk sac edema first appeared at 96 hpf in the 0.02 mg/L treatment group (Figure 1A,B). Higher concentrations induced earlier and more severe effects (Figure 1C,D). At 0.2 mg/L, pericardial edema, yolk sac edema, and abnormal yolk sac morphology were detectable by 72 hpf, while in the 2 mg/L group, malformations were detectable as early as 48 hpf. The incidence and severity of deformities increased concentration-dependently, progressing to include spinal curvature and tail bending, often in combination (Figure 1E,F). No malformations were observed in the control group or in embryos exposed to 0.002 mg/L F-53B (Figure 1G,H).

Hatching success was assessed as a critical developmental endpoint following F-53B exposure. At 48 hpf, hatching rates in the control group exceeded 90%. Similarly, embryos exposed to 0.002 mg/L and 0.02 mg/L exhibited hatching rates above 90%, with no significant differences from the control (*p* > 0.05), indicating that these concentrations do not impair hatching. In contrast, exposure to higher concentrations caused a marked and significant reduction in hatching success. Hatching rates declined to 87.77 ± 1.89% at 0.2 mg/L and 75.33 ± 1.86% at 2 mg/L (*p* < 0.05), demonstrating a concentration-dependent inhibitory effect with a threshold between 0.02 mg/L and 0.2 mg/L.

**Figure 2:**
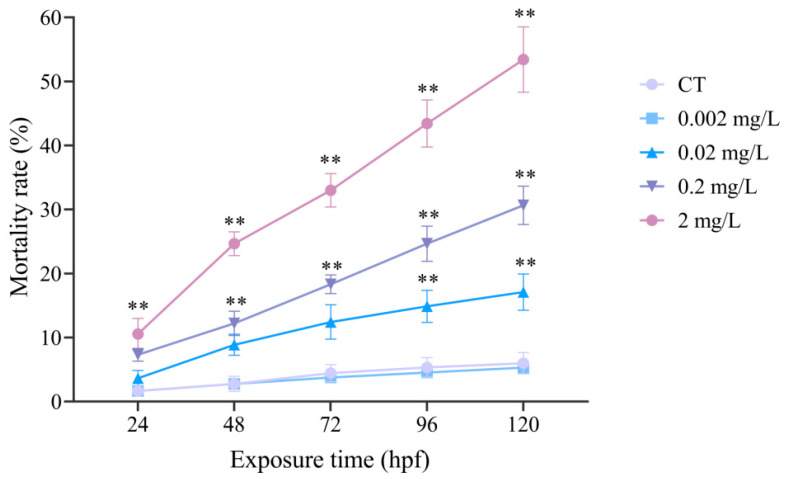
Effects of F-53B on the mortality of *C. maculata* embryos/larvae following 120 h of exposure. Data are presented as mean ± SD (*n* = 3 independent replicates, each comprising a pooled sample of 300 embryos). ** *p* < 0.01.

Lethal effects were assessed over the exposure period. During the first 96 hpf, cumulative mortality remained below 50% in all treatment groups, precluding the calculation of LC_50_ values for the 24–96 h time intervals. However, at 120 hpf, a clear concentration-dependent increase in mortality was observed. Mortality rates reached 17.11 ± 2.84%, 30.67 ± 3.00%, and 53.44 ± 5.10% at 0.02, 0.2, and 2 mg/L, respectively, all significantly higher than the control (6.00 ± 1.67%, *p* < 0.05). Mortality in the 0.002 mg/L group (5.33 ± 0.33%) did not differ from the control (*p* > 0.05), suggesting this concentration is below the lethal effect threshold (Figure 2). Based on 120 hpf mortality data, the estimated LC_50_ was 1.43 mg/L (95% confidence interval: 1.032~2.105 mg/L), providing a quantitative measure of lethal potency in *C. maculata* embryos/larvae.

### 3.2. Histopathological Alterations in the Liver and Intestines

**Figure 3:**
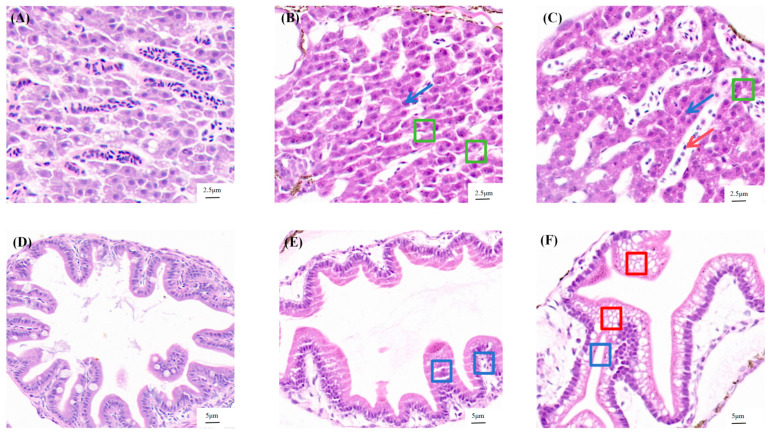
Histopathological alterations in the liver (**A**–**C**) and intestines (**D**–**F**) of *C. maculata* larvae at 120 hpf following F-53B exposure. Representative images are shown for the control (CT), 0.2 mg/L, and 2 mg/L treatment groups. In the liver, green boxes indicate vacuolization, blue arrows denote karyolysis, and red arrows indicate pyknosis. In the intestine, blue boxes highlight villus atrophy, and red boxes mark cellular damage.

Histopathological examination revealed distinct structural alterations in the liver and intestines of exposed larvae. In control larvae, hepatic tissue displayed normal architecture, with regularly arranged, polygonal hepatocytes containing spherical nuclei and clearly defined cell membranes (Figure 3A). In contrast, larvae exposed to 0.2 and 2 mg/L F-53B for 120 h exhibited significant hepatic damage, characterized by disorganized hepatocytes with blurred boundaries, nuclear condensation, and severe vacuolar degeneration (Figure 3B,C). Similarly, intestinal villi in control larvae were intact, lined by regularly arranged columnar epithelial cells with a distinct brush border (Figure 3D). Larvae treated with 0.2 and 2 mg/L F-53B showed marked intestinal compromise, featuring villi atrophy and a loss of epithelial integrity (Figure 3E,F).

### 3.3. Oxidative Stress Biomarker Analysis

**Figure 4:**
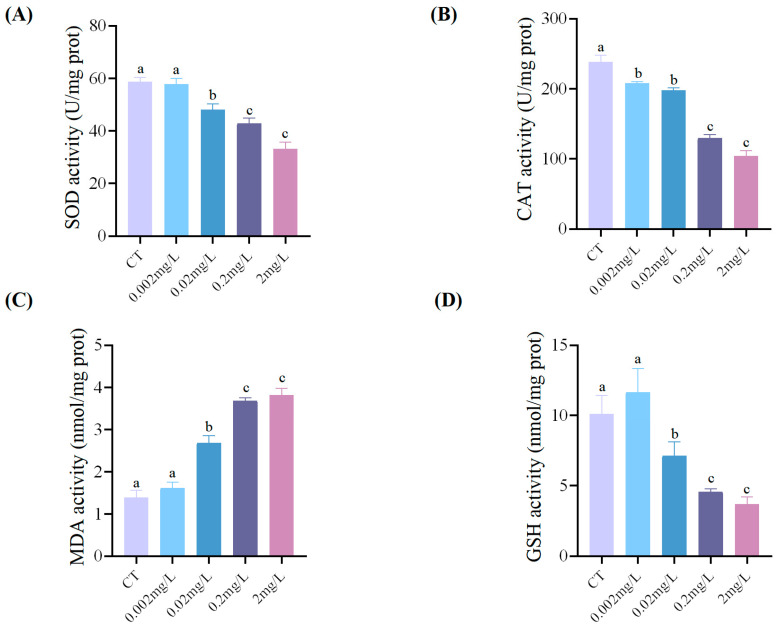
Activities of (**A**) SOD, (**B**) CAT, (**C**) MDA, and (**D**) GSH in *C. maculata* larvae exposed to varying concentrations of F-53B for 120 h. Data are mean ± SD (*n* = 3 independent replicates, each comprising a pooled sample of 15 larvae). Different lowercase letters denote statistically significant differences among groups (*p* < 0.05).

After 120 h of exposure, F-53B induced concentration-dependent alterations in oxidative stress biomarkers. The activities of the antioxidant enzymes SOD and CAT declined progressively with increasing F-53B concentration. SOD activity was significantly reduced at concentrations ≥0.02 mg/L, showing decreases of 17.95%, 26.96%, and 43.38% at 0.02, 0.2, and 2 mg/L, respectively, compared to the control (*p* < 0.05) (Figure 4A). CAT activity was significantly suppressed even at the lowest concentration, declining by 12.65%, 17.00%, 45.46%, and 56.36% across the 0.002, 0.02, 0.2, and 2 mg/L F-53B treatments (*p* < 0.05) (Figure 4B). Conversely, MDA increased markedly, with content rising significantly by 91.95%, 164.18%, and 173.99% at concentrations ≥0.02 mg/L (*p* < 0.05) (Figure 4C). GSH content showed a non-significant increase at 0.002 mg/L (*p* > 0.05) but decreased significantly at higher concentrations, by 29.48%, 54.66%, and 63.45% at 0.02, 0.2, and 2 mg/L, respectively (*p* < 0.05) (Figure 4D).

### 3.4. Transcriptomic Analysis

Transcriptomic sequencing was performed on 15 samples (SRA Accession No. PRJNA1417481). Following quality control, 89.66 Gb of high-quality clean reads were retained, averaging 5.98 Gb per sample, with Q30 scores of 91.91~95.52% and GC content of 40.80~45.04%. Alignment to the *C. maculata* reference genome (SRA Accession No. PRJNA730430) [44] was successful, with mapping rates of 90.5~93.0% (Table 2). A total of 25,284 transcripts were identified, comprising 24,115 annotated protein-coding genes and 1169 novel transcripts. Correlation and principal component analysis (PCA) revealed clear separation between treatment groups and tight clustering of biological replicates within groups, confirming high reproducibility (Figure S1).

**Figure 5:**
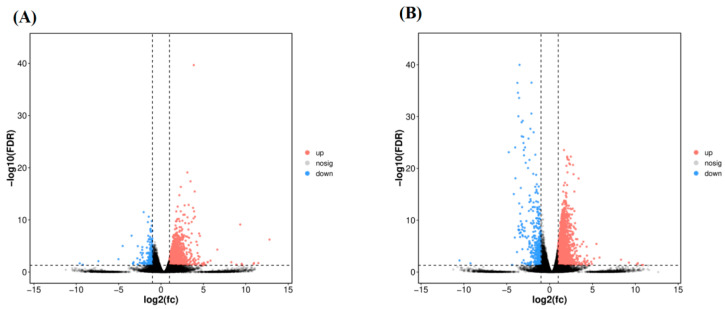
Volcano plots of DEGs for comparisons (**A**) CT vs. 0.2 mg/L F-53B and (**B**) CT vs. 2 mg/L F-53B, fc = fold change.

Differential expression analysis (FDR < 0.05) showed a concentration-dependent increase in the number of DEGs. Compared to the control, DEGs counts increased from 85 (78 up-regulated, 7 down-regulated) in the 0.002 mg/L group and 339 (164 up-regulated, 175 down-regulated) in the 0.02 mg/L group to 1762 (1563 up-regulated, 199 down-regulated) in the 0.2 mg/L group and 2786 (2385 up-regulated, 401 down-regulated) in the 2 mg/L group, with up-regulated genes predominating at all concentrations (Figure 5A,B, Figure S2A,B). Vitellogenin (*vtg2*) was the only DEG common to all exposure groups, while 1194 DEGs were shared between the 0.2 mg/L and 2 mg/L treatments (Figure S2C).

GO enrichment analysis revealed functional shifts across concentrations. Enriched terms in the lower concentration groups (0.002 and 0.02 mg/L) were limited and included categories related to metabolism and basic cellular processes (Figure S3A,B). In contrast, higher concentrations (0.2 and 2 mg/L) induced robust enrichment in immune- and development-related functions (Figure S3C,D). Consistently enriched Biological Process terms included cytokine production (GO:0001816) and regulation of immune system process (GO:0002682). Key Molecular Function terms comprised immune receptor activity (GO:0140375) and cytokine receptor activity (GO:0004896). Commonly enriched Cellular Component terms involved cell projection (GO:0042995) and axon (GO:0030424).

**Figure 6:**
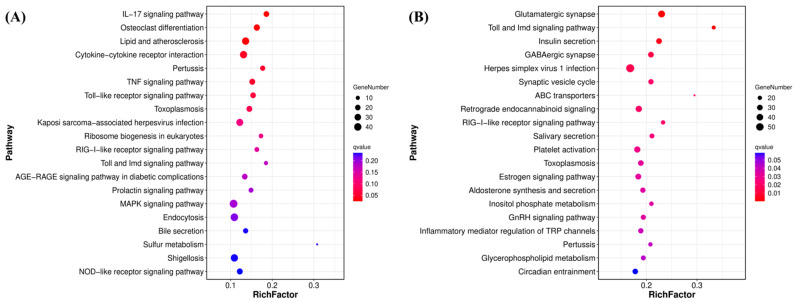
KEGG pathway enrichment analysis. Bubble plots for comparisons: (**A**) CT vs. 0.2 mg/L and (**B**) CT vs. 2 mg/L F-53B. The top 20 pathways with the smallest FDR were selected for visualization. The vertical axis lists pathway names, and the horizontal axis represents the enrichment factor (ratio of DEGs in a pathway to the total genes assigned to that pathway). Bubble size corresponds to gene count, and color intensity indicates statistical significance, with redder hues representing smaller FDR.

KEGG pathway analysis further highlighted a concentration-dependent shift from metabolic to immune-inflammatory responses. The lowest concentration (0.002 mg/L) primarily enriched metabolic pathways such as Bile secretion (ko04976), Chemical carcinogenesis-DNA adducts (ko05204), and Drug metabolism-cytochrome P450 (ko00982) (Figure S4A). In contrast, higher concentrations triggered immune and inflammatory responses. The comparison for CT vs. 0.02 mg/L showed enrichment in IL-17 signaling pathway (ko04657), Osteoclast differentiation (ko04380), Toll-like receptor signaling pathway (ko04620), and TNF signaling pathway (ko04668) (Figure S4B). A similar profile was observed for CT vs. 0.2 mg/L, which included these same pathways plus cytokine–cytokine receptor interaction (ko04060) (Figure 6A). Additionally, the CT vs. 2 mg/L comparison revealed enrichment in other immune and signaling pathways such as Toll and Imd signaling (ko04624), RIG-I-like receptor signaling (ko04622), GnRH signaling (ko04912), and Inflammatory mediator regulation of TRP channels (ko04750) (Figure 6B). Collectively, these results demonstrate that F-53B exposure disrupts genes associated with immune function, inflammatory response, endocrine signaling, and cellular metabolism.

### 3.5. Effect of F-53B on the Expression of Immune-Related Genes

Based on transcriptomic screening DEGs across all experimental groups, eight genes associated with immune processes, *IL-1β*, *IL-8*, *IL-17*, *NF-κb*, *TNF-α*, *IL-10*, *CCR6*, and *CXCR*3, were identified. These genes are involved in pathways including IL-17 signaling pathway, cytokine–cytokine receptor interaction, Toll-like receptor signaling pathway, and TNF signaling pathway. Transcriptomic analysis revealed a concentration-dependent shift in their expression. At 0.002 mg/L F-53B, all eight genes were down-regulated, with *IL-1β*, *IL-8*, *TNF-α*, *IL-10*, and *CCR6* showing significant suppression (*p* < 0.05). Expression levels began to increase at 0.02 mg/L, though *IL-1β*, *IL-8*, and *IL-10* remained significantly lower than controls (*p* < 0.05). At 0.2 mg/L, a clear up-regulatory trend emerged, with *IL-1β*, *IL-10*, *IL-17*, *CXCR*3 and *CCR6* becoming significantly elevated (*p* < 0.05). Finally, exposure to 2 mg/L F-53B resulted in the marked up-regulation of all eight genes (*p* < 0.05). These results indicate that higher concentrations (≥0.2 mg/L) significantly up-regulate key genes within critical immune-inflammatory pathways (Figure S5).

**Figure 7:**
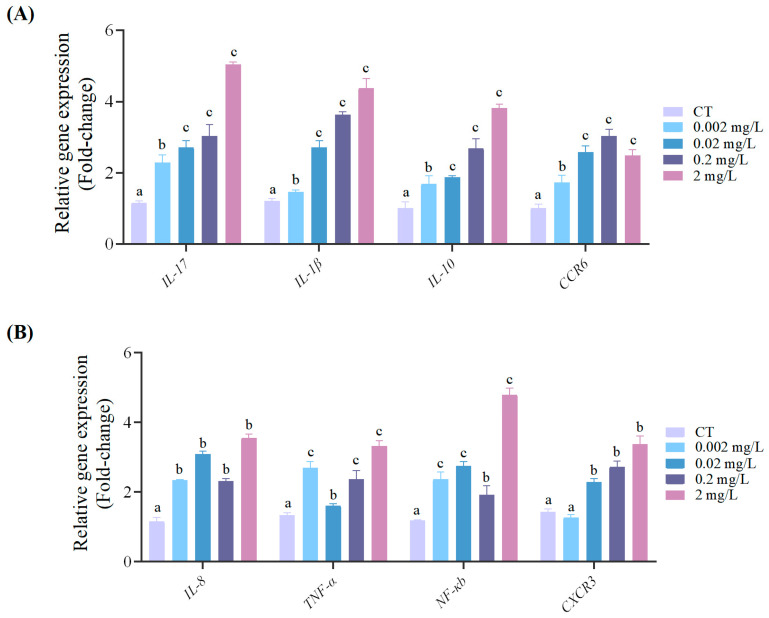
F-53B exposure modulates the expression immune- and inflammation-related genes (**A**,**B**) in *C. maculata* larvae. Expression profiles are shown across a concentration gradient of F-53B (CT, 0.002, 0.02, 0.2 and 2 mg/L). Letters above bars denote statistically significant differences among treatments (*p* < 0.05).

These transcriptomic findings were validated by qRT-PCR using the same concentration gradient. Consistent with the sequencing data, the expression of *IL-17*, *IL-1β*, and *IL-10* increased significantly at concentrations above 0.02 mg/L (*p* < 0.05). *CCR6* expression rose with increasing F-53B concentrations up to 0.2 mg/L. Although a slight decrease was observed at 2 mg/L, its expression remained significantly elevated relative to the control (*p* < 0.05) (Figure 7A). The expression levels of *IL-8*, *TNF-α*, and *NF-κb* varied across the gradient but peaked significantly at 2 mg/L (*p* < 0.05). *CXCR*3 expression decreased slightly at 0.002 mg/L F-53B, though not significantly compared with the control (*p* > 0.05), then increased progressively, reaching a significant peak at 2 mg/L (*p* < 0.05) (Figure 7B).

## 4. Discussion

In this study, we investigated the developmental toxicity of F-53B in *C. maculata* embryos/larvae using a concentration range (0.002–2 mg/L) designed to bridge ecological relevance and mechanistic understanding. Environmental monitoring data indicate that F-53B concentrations in surface waters generally range from below the limit of quantification to 50 ng/L in receiving waters distant from point sources, with levels up to 968 ng/L (~0.001 mg/L) detected in waters adjacent to electroplating facilities. Elevated concentrations have also been reported in chrome plating wastewater (43~112 μg/L). The lowest concentration tested in our study (0.002 mg/L, equivalent to 2 μg/L) therefore falls within the upper range of environmentally relevant levels, particularly in areas influenced by industrial discharge, such as the Pearl River Basin [15,19]. The intermediate concentrations (0.02 and 0.2 mg/L) were selected to capture sublethal responses and establish concentration–response gradients, while the highest concentration (2 mg/L) was chosen based on preliminary range-finding tests to remain below the acute lethality threshold (complete mortality at ≥5 mg/L) yet still elicit measurable phenotypic and molecular effects. This tiered design acknowledges that while effects at 0.2~2 mg/L exceed typical ambient levels, such concentrations may occur in localized hotspots or accidental release scenarios, and are essential for elucidating toxicity mechanisms and deriving hazard data for ecological risk assessment.

Exposure to F-53B adversely affected the survival and development of *C. maculata* embryos/larvae. Higher concentrations (0.2 and 2 mg/L) significantly reduced embryo hatching rates, whereas lower concentrations (0.002 and 0.02 mg/L) had minimal impact. This response appears species-specific, differing from observations in *D. rerio*, in which F-53B primarily delays hatching timing rather than reducing final hatching rates, with nearly all embryos hatching by 72 hpf even at 12 mg/L [42]. In *C. maculata,* larval mortality increased concentration-dependently and occurred earlier at higher doses. Similarly, the onset and severity of malformations, such as yolk sac edema, pericardial edema, spinal curvature, and tail bending, were both earlier and more pronounced with increasing concentration. These findings align with the reported developmental toxicity of F-53B in other fish. For example, while low concentrations (≤0.3 mg/L) did not significantly affect survival or body length in *D. rerio* larvae [14], higher doses (6 and 12 mg/L) reduced survival and increased malformations [42]. Furthermore, exposure to 0.2 mg/L F-53B for 5 days reduced body weight in *D. rerio* larvae, an effect that persisted after depuration [31]. Collectively, the results indicate that F-53B induces concentration-dependent developmental toxicity, potentially compromising fish health through impaired survival, growth, and morphogenesis.

Given the structural similarity between F-53B and PFOS, a compound known to induce hepatotoxicity, neurotoxicity, immunotoxicity, and reproductive and developmental toxicity [6,7,8,9], F-53B is hypothesized to exhibit comparable toxic effects, yet its tissue-level impacts require experimental investigation. Exposure to 0.2 and 2 mg/L F-53B caused significant hepatic injury in *C. maculata* larvae, characterized by hepatocellular vacuolization and nuclear pyknosis. These histopathological changes are consistent with reports in other species. In *M. musculus*, F-53B exposure increased liver weight and caused histopathological damage, including apoptosis, lipid accumulation, steatosis, and necrosis [47]. Comparable hepatic effects have been observed in fish, such as marked hepatocellular vacuolization in adult hainan medaka (*Oryzias curvinotus*) [48], and enhanced vacuolization with apoptosis in GIFT tilapia (*Oreochromis niloticus*) [49]. The observed vacuolization may result from lipid infiltration due to disrupted metabolism, while cytoplasmic lysis and nuclear pyknosis are indicative of apoptotic or necrotic hepatocyte death [50]. Furthermore, exposure to 2 mg/L F-53B for 120 h induced intestinal villi atrophy and loss of epithelial integrity in *C. maculata* larvae. Similar detrimental effects on intestinal structure have been reported in other species, including impaired barrier function and inflammation in *M. musculus* [51], reduced villus height in *D. rerio* [52], and villi erosion and fusion in *O. curvinotus* [48]. This structural damage may be attributable to gut barrier dysfunction and inflammatory responses [51].

Tissue damage rapidly activates the antioxidant defense system. Within this system, SOD and CAT constitute the primary enzymatic defense. Under normal physiological conditions, ROS production and the scavenging capacity of these enzymes remain in dynamic equilibrium, thereby maintaining redox homeostasis [49]. When ROS levels increase, SOD converts superoxide anions into hydrogen peroxide (H_2_O_2_), which is then decomposed by CAT into water and oxygen [53]. Typically, SOD activation is often accompanied by up-regulation of CAT activity, providing coordinated protection against oxidative damage. However, when this system is overwhelmed, excess ROS can lead to enzyme inhibition, inactivation, or degradation, increasing the risk of oxidative damage [39]. Pollutants such as F-53B have been shown to induce oxidative stress in aquatic organisms, including *D. rerio* embryos/larvae [54]. In this study, exposure of *C. maculata* embryos/larvae to 0.2 and 2 mg/L F-53B significantly reduced both SOD and CAT activities, likely due to enzyme inhibition or inactivation caused by excessive ROS production. A concurrent decrease in GSH further indicated compromised antioxidant capacity. Moreover, elevated levels of MDA, a terminal product of lipid peroxidation [55], were observed in 120 hpf *C. maculata* larvae following high-concentration F-53B exposure, suggesting excessive ROS generation and pronounced lipid peroxidation. Collectively, the decline in SOD and CAT activities, GSH depletion, and increased MDA demonstrate that high-concentration F-53B induces severe oxidative stress during early development in *C. maculata*, which may contribute to the developmental abnormalities observed in this species.

The immunotoxicity of environmental pollutants serves as a key indicator of ecological risk. In fish, cytokines, including interleukins, chemokines, and tumor necrosis factors, play a central role in maintaining immune homeostasis, and their dysregulation is closely linked to inflammatory responses [14,39]. Pro-inflammatory markers such as *IL-17*, *IL-1β*, *IL-8*, and *TNF-α* are known to drive inflammation [56], whereas the anti-inflammatory cytokine *IL-10* contributes to intestinal homeostasis, apoptosis inhibition, and immune balance [57,58]. Previous studies have demonstrated that F-53B up-regulates these cytokines in *D. rerio* embryos and larvae [14,59], with altered expression of the chemokine receptors *CCR6* and *CXCR3* further indicating inflammatory activation [14]. In the present study, similar responses were observed in 120 hpf *C. maculata* larvae, suggesting that F-53B may also disrupt immune function in this species.

Mechanistically, F-53B exerted concentration-dependent immunomodulatory effects. Lower concentrations primarily perturbed metabolic pathways, including bile secretion and drug metabolism, whereas higher exposures were associated with enrichment of immune- and inflammation-related pathways. GO enrichment analysis revealed significant over-representation of terms related to cytokine production, immune system regulation, and immune receptor activity. KEGG pathway analysis further identified alterations in TLR, TNF, and MAPK signaling pathways, which are recognized regulators of inflammatory responses. The TLR pathway initiates immune activation [60], and its downstream MAPK cascade may amplify the expression of pro-inflammatory cytokines such as *IL-1β*, *IL-8*, and *TNF-α* [59]. These pathways have been implicated in immune toxicity regulation in *D. rerio* [59,61]. In this study, up-regulation of key genes within these pathways (*IL-1β*, *TNF-α*, *IL-8*, *NF-κb*) coincided with antioxidant system activation and inflammatory induction, suggesting involvement of the TLR-MAPK axis in F-53B-induced immune dysregulation. These transcriptomic findings were corroborated by qRT-PCR, which showed significant up-regulation of *IL-1β*, *IL-8*, *TNF-α*, *IL-17*, *IL*-10, *CCR6*, and *CXCR3* at higher F-53B concentrations (0.2 and 2 mg/L), consistent with enrichment of IL-17 and TNF signaling pathways. Collectively, these results indicate that beyond a certain concentration threshold, F-53B shifts the physiological response from metabolic adaptation toward inflammatory signaling, potentially contributing to oxidative stress, inflammation, and disruption of immune homeostasis in early-stage *C. maculata* via mechanisms that may involve the TLR-MAPK cascade.

**Figure 8:**
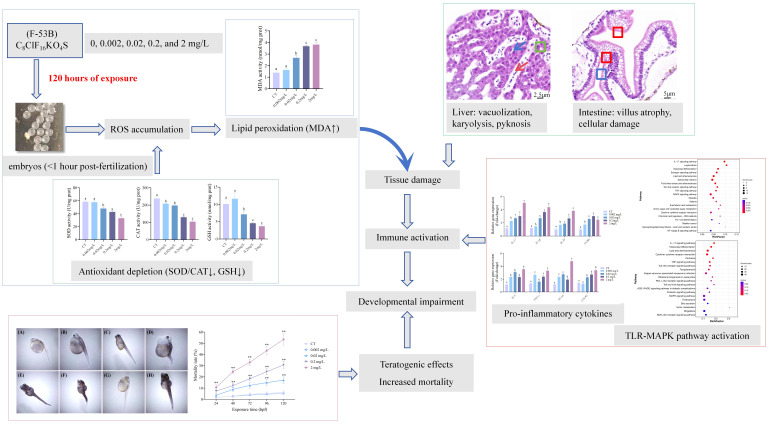
Proposed mechanistic pathway of F-53B-induced developmental toxicity in *C. maculata* embryos/larvae. This schematic integrates findings from biochemical, histopathological, and molecular analyses. Arrows indicate sequential events: F-53B exposure → ROS accumulation → antioxidant depletion → lipid peroxidation → tissue damage → immune activation → developmental impairment. Blue boxes represent upstream oxidative stress responses, green boxes represent intermediate tissue damage, and red boxes denote downstream outcomes, including immune dysregulation and developmental toxicity.

In general, this study provides valuable preliminary insights into the developmental toxicity of F-53B on *C. maculata* embryos/larvae. Exposure to F-53B disrupts redox homeostasis, leading to ROS accumulation and antioxidant depletion. This oxidative stress induces lipid peroxidation, compromising membrane integrity and causing tissue damage in the liver and intestines. In response, damaged tissues up-regulate pro-inflammatory cytokines and may activate the TLR-MAPK pathway, triggering an inflammatory response that ultimately drives developmental toxicity, manifested as malformations and elevated mortality (Figure 8). However, we acknowledge that the use of nominal concentrations without analytical verification represents a methodological limitation. Factors such as adsorption to glass aquaria and solvent interactions were not assessed, which may affect exposure accuracy. Although consistent concentration-dependent trends across multiple endpoints support the reliability of our findings, the absence of measured concentrations constrains the precision of effect thresholds. In future studies, we will incorporate analytical methods such as LC-MS/MS to verify exposure concentrations and further strengthen the robustness of our toxicological assessments.

## 5. Conclusions

This study systematically evaluated the physiological, molecular, and histological effects of F-53B on *C. maculata* embryos/larvae. Results revealed that F-53B exposure induced concentration-dependent developmental toxicity in *C. maculata*, characterized by reduced hatching rates, increased mortality, and morphological malformations (e.g., yolk sac edema, spinal curvature). Histopathological analysis indicated significant hepatic injury (vacuolization, nuclear pyknosis) and intestinal damage (villi atrophy) at higher concentrations (0.2 and 2 mg/L). Mechanistically, F-53B exposure was associated with oxidative stress, evidenced by suppressed SOD and CAT activities, GSH depletion, and elevated MDA levels. Immune-related effects were also observed, including the up-regulation of pro-inflammatory cytokines such as *IL-1β*, *IL-8*, and *TNF-α*, consistent with involvement of the TLR-MAPK signaling pathway. These findings suggest a shift from metabolic processes toward inflammatory responses at higher exposure levels. Collectively, the data indicate that F-53B may impair early development in *C. maculata* through pathways involving oxidative damage, tissue injury, and immune disruption. Future research should integrate multi-omics approaches with chronic exposure studies to better understand the ecological implications of F-53B and support environmental risk assessment.

## Figures and Tables

**Table 1:** Primer sequences for qRT-PCR.

Gene Name	Forward Primer (5′-3′)	Reverse Primer (5′-3′)
*CCR6*	CATCGCAGACCTGCTGTTTG	TGCAGATGATGCGGCTGTAA
*CXCR3*	CCTTGCTTGAGGGCCTTGATA	CCATTCCCAAGGACACCCAC
*TNF-α*	ACAATACCACCCCAGGTCCCA	ACGCAGCATCCTCTCATCCAT
*IL-8*	CTATTGTGGTGTTCCTGA	TCTTCACCCAGGGAGCTTC
*IL-10*	CAGTGCAGAAGAGTCGACTGCAAG	CGCTTGAGATCCTGAAATATA
*IL-17*	GTCTCTGTCACCGTGGAC	TGGGCCTCACACAGGTACA
*IL-1β*	GTTTACCTGAACATGTCGGC	AGGGTGCTGATGTTCAGCCC
*NF-κb*	CAGCCAAAACCAAGAGGGAT	TCGGCTTCGTAGTAGCCATG
*β-actin*	TTGAGCAGGAGATGGGAACCG	AGAGCCTCAGGGCAACGGAAA

**Table 2:** Summary statistics for the sequencing data derived from 15 transcriptome libraries.

Sample	RawDatas	CleanData (%)	Q30 (%)	Total Mapped (%)	Sequenced Total Genes (%)
CT-1	40,424,646	99.66%	94.98%	91.12%	85.52%
CT-2	45,328,962	99.58%	95.38%	91.09%	83.19%
CT-3	46,435,866	99.57%	95.35%	91.40%	82.81%
0.002-1	40,545,500	99.58%	95.07%	91.78%	84.46%
0.002-2	46,899,806	99.32%	91.91%	90.51%	85.30%
0.002-3	40,622,042	99.56%	94.71%	92.68%	86.27%
0.02-1	40,067,762	99.61%	94.94%	92.24%	85.13%
0.02-2	42,448,750	99.60%	95.28%	92.55%	85.01%
0.02-3	39,142,266	99.61%	94.96%	92.33%	82.69%
0.2-1	46,590,220	99.58%	95.32%	92.85%	84.28%
0.2-2	46,920,306	99.65%	95.51%	92.75%	85.05%
0.2-3	45,471,018	99.58%	95.44%	92.72%	84.39%
2-1	40,973,028	99.61%	95.05%	92.59%	84.84%
2-2	41,708,338	99.61%	95.24%	92.42%	84.25%
2-3	46,891,486	99.66%	95.52%	93.00%	85.71%

## Data Availability

The original data presented in this study are publicly available in the NCBI database at https://www.ncbi.nlm.nih.gov/guide/data-software/ (accessed on 3 March 2026), with accession No. PRJNA1417481.

## Supplementary Materials

The following supporting information can be downloaded at: https://www.mdpi.com/article/10.3390/antiox15030368/s1.
