# Supplementary material for: Developmental Toxicity of Chlorinated Polyfluorinated Ether Sulfonate (F-53B), a Perfluorooctane Sulfonate (PFOS) Alternative, in Embryos and Larvae of Blotched Snakehead (Channa maculata)
Source: Antioxidants (Basel). 2026 Mar 13;15(3):368. doi: 10.3390/antiox15030368 (PMC13024560; doi:10.3390/antiox15030368)
Supplement: Supplementary file 1 [file antioxidants-15-00368-s001.zip › antioxidants-4159497-supplementary.pdf]

Supplementary Materials:

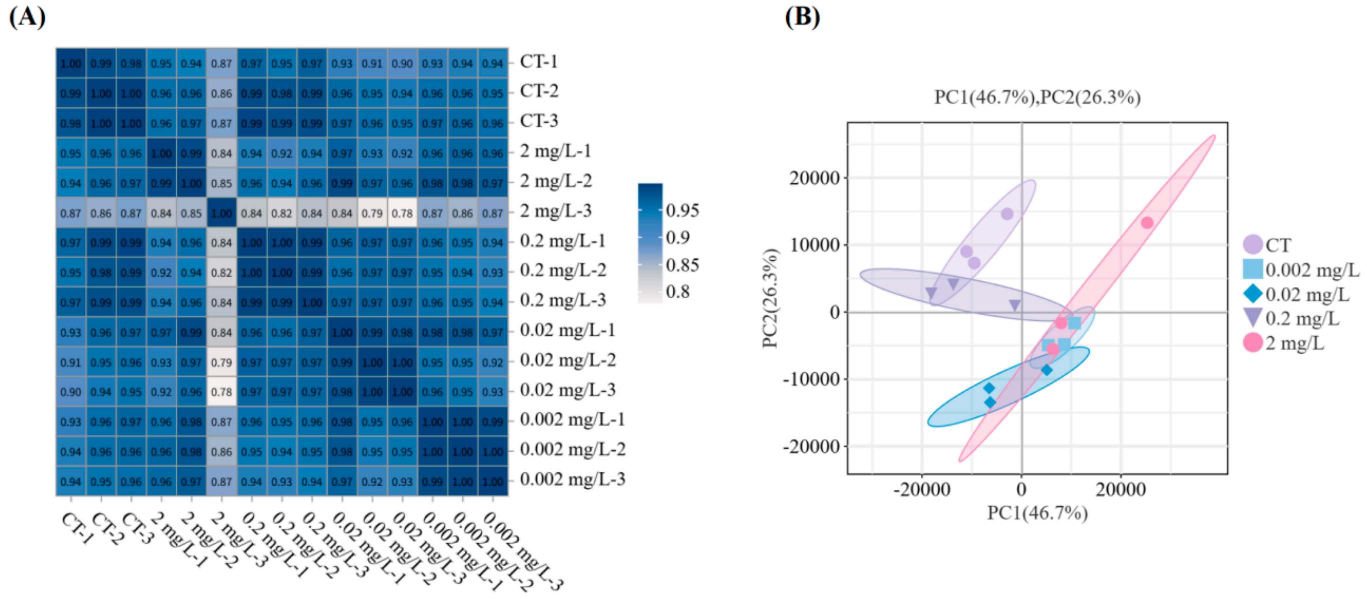

Figure S1. (A) Sample correlation heatmap and (B) principal component analysis plot..

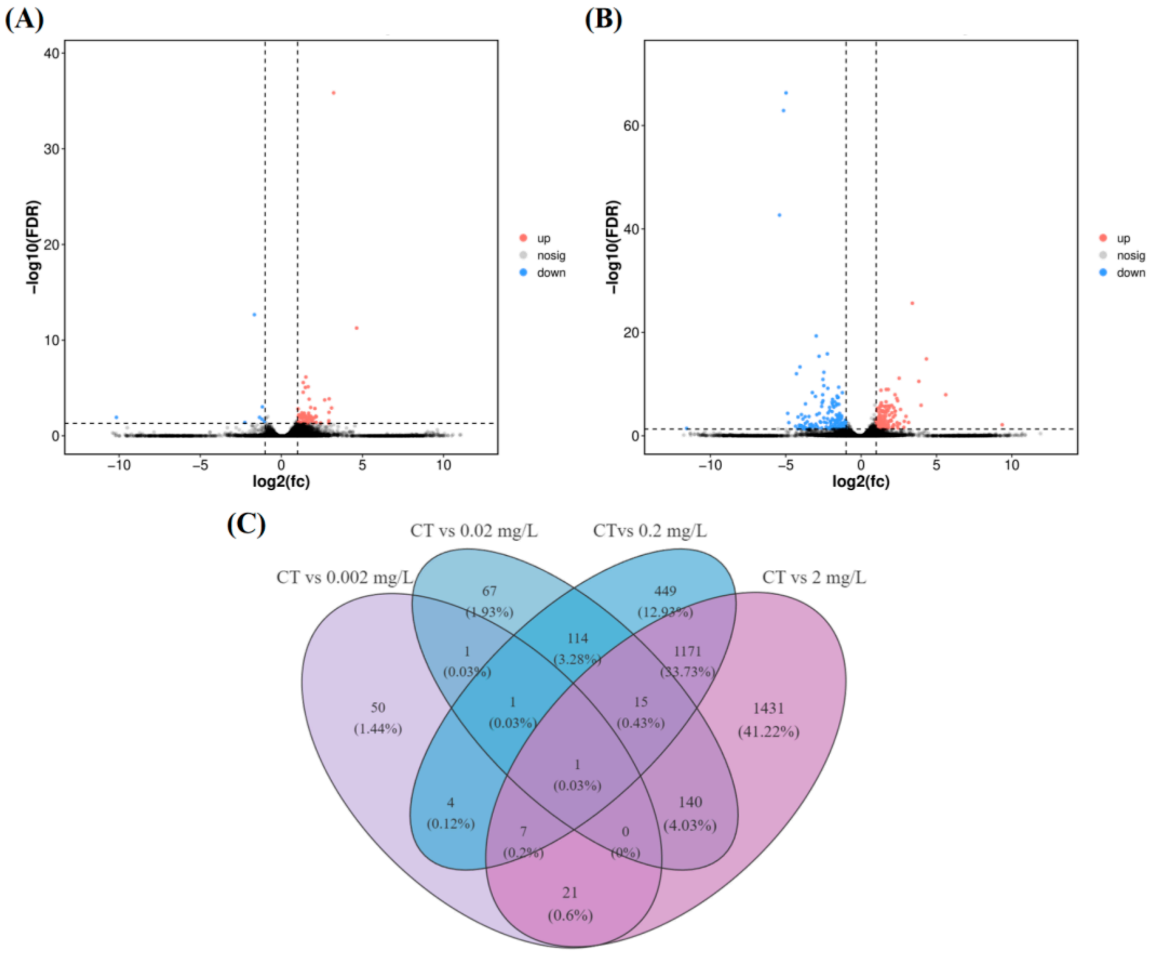

Figure S2. Volcano plots of DEGs for comparisons (A) CT vs. 0.002 mg/L and (B) CT vs. 0.02 mg/L F-53B, (C) Venn diagram of the overlapped DEGs across all treatment groups compared to the control (CT).

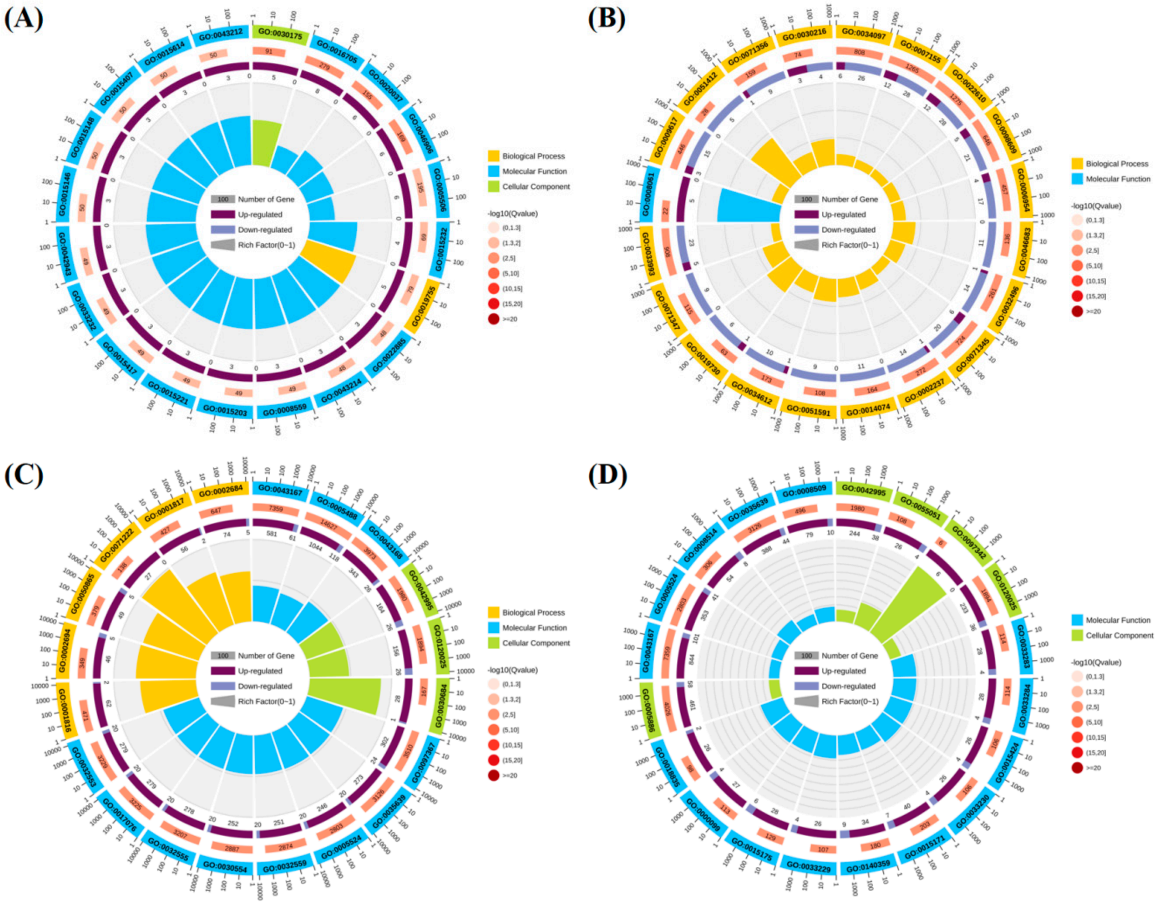

Figure S3. Circle diagram of GO enrichment analysis for comparisons (A) CT vs. 0.002 mg/L, (B) CT vs. 0.02 mg/L, (C) CT vs. 0.2 mg/L, and (D) CT vs. 2 mg/L F-53B. The outermost ring displays the top 20 enriched GO terms, color-coded by ontology category (biological process, cellular component, molecular function) with an associated scale for DEG count. The second ring uses bar length to represent gene count per term, with color intensity corresponding to the FDR, with deeper red indicating greater significance. The third ring displays the proportion of up-regulated (deep purple) and down-regulated (light purple) DEGs, with exact percentages labeled. The innermost ring plots the Rich Factor for each term, calculated as the ratio of DEGs to total genes in that GO term, against a background grid (unit = 0.1).

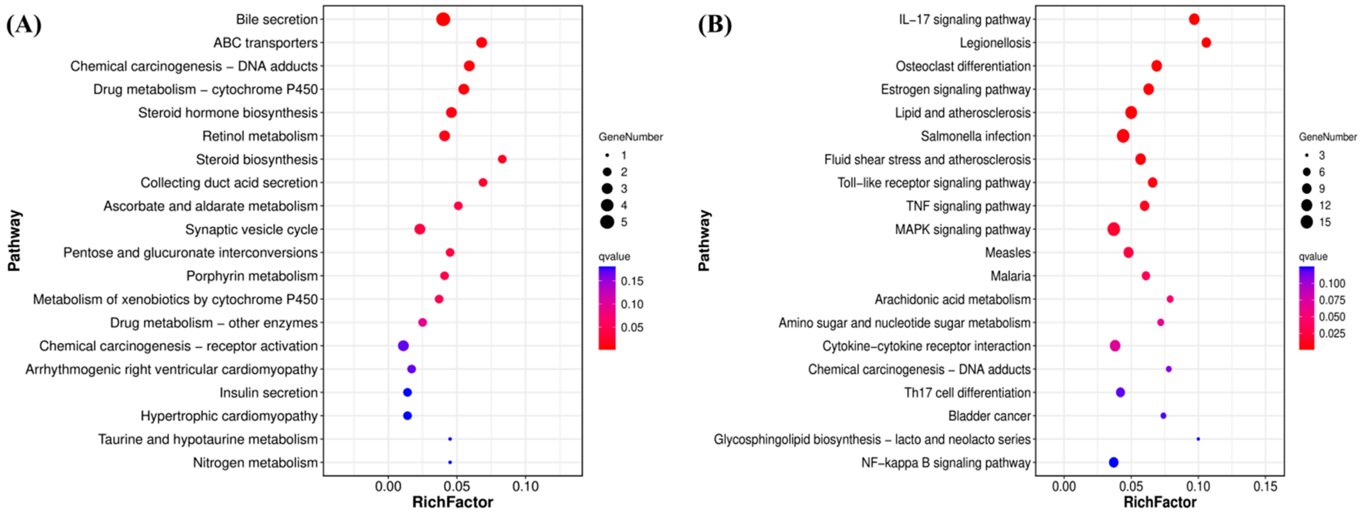

Figure S4. KEGG pathway enrichment analysis. Bubble plots for comparisons (A) CT vs. 0.002 mg/L, (B) CT vs. 0.02 mg/L.The top 20 pathways with the smallest FDR were selected for visualization. The vertical axis lists pathway names, and the horizontal axis represents the enrichment factor (ratio of DEGs in a pathway to the total genes assigned to that pathway). Bubble size corresponds to gene count, and color intensity indicates statistical significance, with redder hues representing smaller FDR.

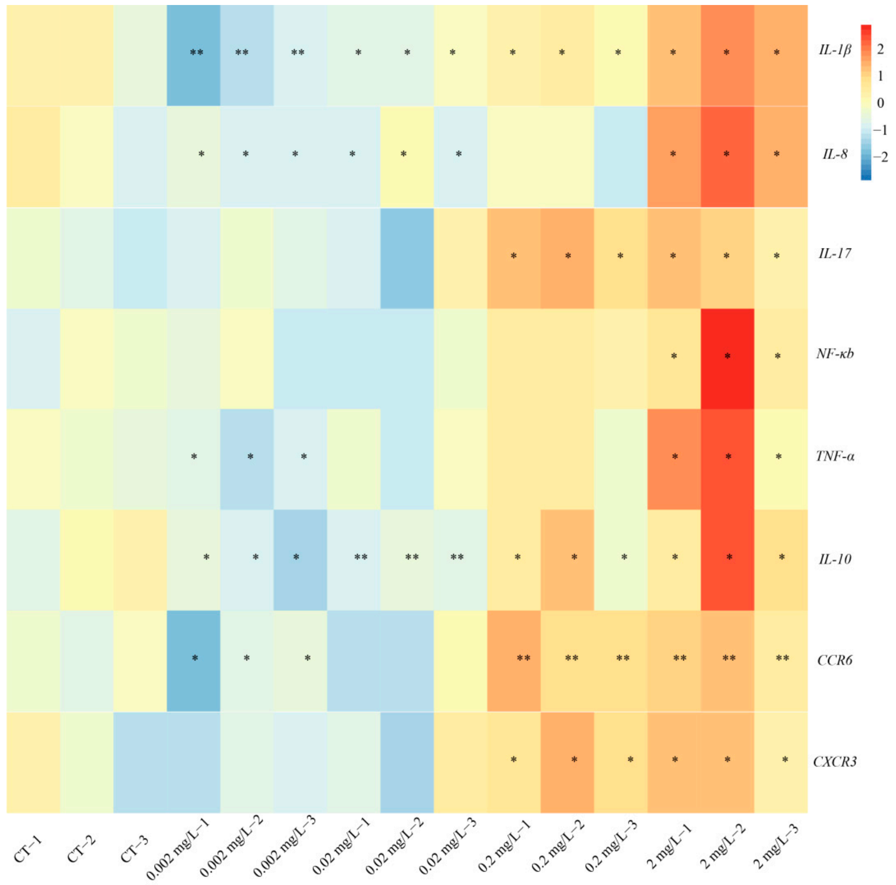

Figure S5. Expression levels of immune-related genes from transcriptomic analysis in 120 hpf *C. maculata* larvae after exposure to F-53B. * indicates statistical significance ($p < 0.05$); ** indicates high statistical significance ($p < 0.01$)
